# Gastrointestinal microbiota of sympatric pipefish (*Syngnathus typhle*) and stickleback (*Gasterosteus aculeatus*) indicate trade-off associated with evolutionary stomach loss

**DOI:** 10.1186/s12862-026-02546-4

**Published:** 2026-06-30

**Authors:** Ralf F. Schneider, Jule Peter, Nils Newrzella, Silke-Mareike Marten, Isabel Tanger, Olivia Roth

**Affiliations:** Marine Evolutionary Biology, Zoological Institute, Kiel University, Kiel, Germany

**Keywords:** Microbiome, Pipefish, Stickleback, *Syngnathus typhle*, *Gasterosteus aculeatus*, Stomach loss

## Abstract

**Background:**

Animal gastrointestinal tracts generally evolved towards a diverse and spatially structured organ system for efficient food digestion. In it, food is chemically broken down and bacterial load reduced by gastric acid in the stomach, acting as a “gatekeeper” for microbes entering the intestines where chyme nutrients and water are absorbed. The natural microbiota across gastrointestinal tract zones support digestion, compete with ingested pathogens and acts itself as an immune stimulus. Despite its important role, several lineages of fish, such as pipefishes, have secondarily lost their stomach and evolved agastric digestion, with unknown consequences to their intestines’ microbiomes.

**Results:**

Here, we test how stomach loss might affect the microbiome by investigating the fore-, mid- and hindgut’s autochthonous microbiota of the Baltic Sea broadnosed pipefish, *Syngnathus typhle*, and comparing it to the stomach, fore- and hindgut’s autochthonous microbiota of the sympatric and ecologically similar three-spine stickleback, *Gasterosteus aculeatus*. Using 16S-rRNA gene sequencing and qPCR, we show that microbial abundance is high in the stomach, accompanied by high alpha diversity, but low in the intestine of *G. aculeatus*, although microbial diversity remains at intermediate levels – a pattern almost inversed in *S. typhle*. *G. aculeatus*’ stomach has the most distinct microbiota across gastrointestinal zones; however, this species’ intestines’ microbes are also found in *S. typhle*. In contrast, the pipefish’s hindgut is the most distinct zone, and many microbes shared across its whole intestine are not found in the *G. aculeatus*.

**Conclusions:**

Our data supports the notion of the stomach and its distinct microbiome being an immunological gatekeeper for the gut, but also suggests that *S. typhle* might benefit from the additional microbes as many indicator taxa are suspected to act as mutualistic symbionts. Stomach-loss may therefore be a trade-off between improved chemical digestion capabilities and an immunological gate-keeper vs. improved microbial digestion and increased immune stimulation.

**Supplementary Information:**

The online version contains supplementary material available at 10.1186/s12862-026-02546-4.

## Background

As heterotrophs, animals must ingest food, break it down both mechanically & chemically, absorb nutrients, and then expel the indigestible components during defecation. In the vast majority of gnathostomes, digestion occurs entirely in the gastrointestinal tract (GIT). Food enters through the mouth, where it is mechanically broken down via mastication and sometimes mixed with saliva and enzymes, facilitating digestion. The food then passes through the oesophagus into a stomach, a dilated structure that commences digestion by mechanically and chemically breaking down food via enzymes and gastric acid [1, 2]. In the subsequent tube-like intestines, nutrients are absorbed via diffusion, active transport and osmosis, while indigestible waste is egested as feces through the anus [2]. This functional separation and specialization in distinct morphological regions of the gastrointestinal tract allows animals to effectively absorb nutrients from their food [2, 3].

Across animal taxa, the digestive system has evolved with considerable variation, often reflecting their specific trophic ecological niches [2–4]. For instance, fish feeding on particularly robust food, such as a plant-rich diet, typically have relatively long intestines, which allow for prolonged digestive processes [5, 6]. In contrast, fish species with a more carnivorous diet only require relatively short intestines as animal tissue is easily digested. Digestion depends not only on the gut morphology and physiology, but also the natural gut microbiota therein: a community of symbiotic bacteria living in the hosts’ intestines and utilizing the provided half-digested food slurry to grow and multiply [3]. Such microbiota can enhance the host’s digestive capabilities by producing enzymes that break down even robust food compounds (e.g., cellulose), which the host otherwise could not digest. This can supplement or even be the hosts’ single-source of essential organic compounds, such as fatty acids or vitamins, which are provided by the bacteria and absorbed by the host [7]. Gut microbiota diversity typically correlates with nutrient yield and supply of essential organic compounds to the host. The members of the gut microbiota are in competition with each other and interact across the full spectrum of symbiosis: from mutualism and commensalism to parasitism [8]. This complex interplay not only stimulates and instructs the host’s immune system vigilance, but also serves as an often-hostile environment for pathogens entering the body via food items [9]. A diverse and undisturbed gut microbiome thus significantly contributes to host health.

Comparative studies show that the morphology and chemical environment shape the microbiota composition along the GIT. For instance, different gut morphologies harbor distinct microbial communities [10–12], while stomach acidity suppresses certain microbes from entering the gut [13]. As such, the rather acidic stomach environment is distinct from the gut environment, featuring bacteria with high resistance to low pH environments. Thus, the stomach is not only an important compartment for nutrient absorption, but also a selective gateway for microbes entering the GIT. Despite these and other advantages of a stomach (see for instance [14]), several gnathostome lineages have evolved agastric digestion with a complete (functional) loss of their stomach. These include sarcopterygians, such as lungfish, and monotremes, and several actinopterygian species, among them zebrafish and medaka, but also fugu, wrasses and all syngnathids (*Syngnathidae*; pipefishes & seahorses), among others [14, 15]. Across these taxa, stomach loss affected not only the morphology, but also the gene repertoire and physiological functions of the body. For instance, it has been reported that agastric fish have lost (often convergently) several genes, including *ghrl*, which regulates appetite, and their losses might be linked to behavioral alterations [14, 16]; via NCBI Blast searches, we also confirmed likely *ghrl* loss here for the agastric taxa: Beloniformes, Tetradontiformes and Syngnathidae).

Many syngnathid lineages, such as seahorses, have markedly altered and typically reduced immune gene repertoires compared to other teleosts [17–20]. Altered immune gene repertoires in this family are thought to have at least partially co-evolved with the unique male pregnancy of this group, allowing for a reduced immune vigilance and thus likelihood of embryo rejection during pregnancy [18–20]. Additionally, several studies in these fish have pointed towards a link between the GIT microbiota and immunity [21–23], however, whether or not the evolutionary loss of the stomach has affected these immunological trajectories remains unclear. Syngnathids do appear to have reduced food digestion capabilities, which consequently resulted in a high food demand and an induced feeding activity throughout the day (as summarized in [15]). This is complemented by adjusted gut evacuation rates according to temperature and food availability for an optimization of nutrient assimilation rates [24], suggesting that stomach loss might not have co-evolved with a more diverse and thus mutualistically more capable gut microbiota. To understand if the syngnathid GIT may still be divided into zones with different digestive functions, the spatial structuring of the microbial community has to be investigated, which has not been done to date (but see [25], for an agastric non-syngnathid species). Such knowledge will allow us to better understand the evolutionary mechanisms and trade-offs underlying stomach loss specifically, and modifications of the GIT more generally.

## Materials

### Aims and study significance

Here, we investigated the GIT of *Syngnathus typhle*, a pipefish lacking (like all syngnathids) a stomach, to describe the spatial structuring of its gut microbiota, and we compared it to the GIT microbiota of a sympatric three-spined stickleback, *Gasterosteus aculeatus*, which does have a stomach. Both species co-occur during their breeding season in shallow eelgrass meadows (although *G. aculeatus* is also common in other habitats), and as similarly sized mesopredators both feed primarily on small marine crustaceans [26]. We hypothesized that the autochthonous (i.e., resident) microbiota communities differ across zones of the GIT in both species and that both species differ in these patterns independent of their food. We further hypothesized that the absence of the stomach in *S. typhle* is associated with an increased microbiota diversity and abundance in downstream gut zones. While we only look at one species pair and do not consider the microbiota attached to food items, our study provides first valuable insights into the role of the stomach as a gate-keeper of the gastrointestinal tract. We emphasize the unique immune-ecological challenges that agastric lineages face, especially in the case of pipefishes – a lineage with an extremely modified immune gene repertoire [18, 20, 20, 27, 28]. However, we also highlight the potential advantages of stomach loss on the mutual gastrointestinal microbiota.

### Fish husbandry and sampling

**Fig. 1:**
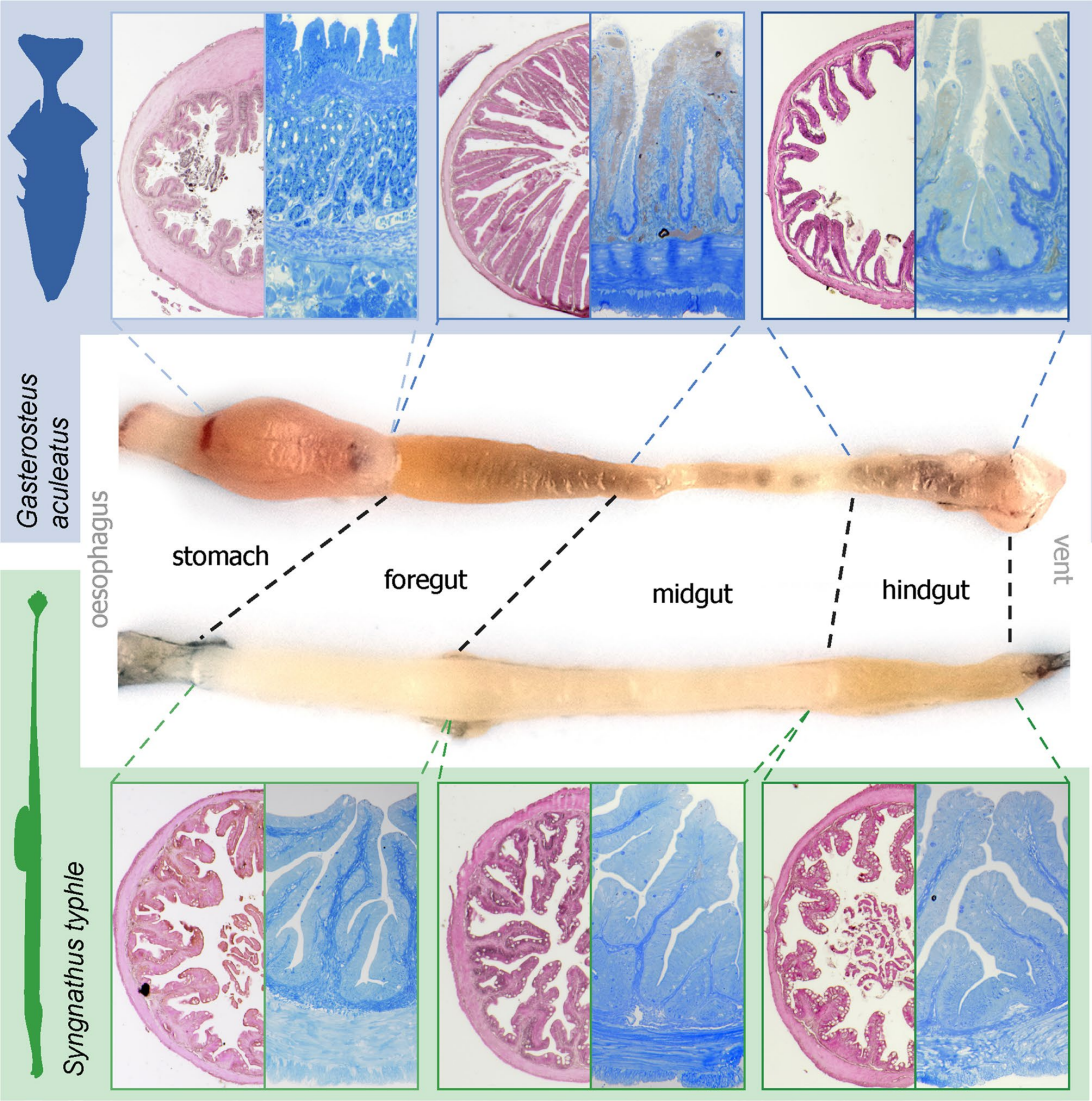
Morphological overview of the gastrointestinal tracts and sampling design. For microbiota analyses, three segments of each species’ gastrointestinal tract were dissected: stomach (only *G. aculeatus*; upper panel), foregut, midgut (only *S. typhle*; lower panel) and hindgut. H&E staining (in reddish) or Richardson’s solution (in bluish) histological sections illustrate gross morphology

For microbiota analyses, a total of 11 *S. typhle* and 11 *G. aculeatus* specimen (each five females & six males) were caught simultaneously in an eelgrass meadow in the Baltic Sea near Orth, Fehmarn (54°26’46.3”N 11°02’26.6”E) and transferred to a flow-through aquarium system with natural Baltic Sea water (12–14 °C, ~15PSU). Sampling these individuals’ GITs immediately would have expectedly led to a microbiome dominated by the allochthonous (transient, food-linked) microbiota. Additionally, we expected that waiting just until post gut evacuation would have deminished this effect only slightly. Instead, fish were kept for six weeks while being fed with frozen mysids and *Artemia* nauplii (i.e., non-local food items), to guarantee both species were confronted with identical food items and thus food-associated microbiota. The day before sampling, fish were not fed to reduce the number of microbes predominantly associated with the diet, and to increase the likelihood of sampling empty GIT segments and thus primarily the autochthonous microbiota. Fish were euthanized using 0.04% MS222 (from 0.4% stocks in PBS), followed by head section in accordance with local ethics regulations (permit issued by the “Ministerium für Landwirtschaft, ländliche Räume, Europa und Verbraucherschutz des Landes Schleswig-Holstein”; No. V242-35168/2018). For *G. aculeatus*, the stomach, fore- and hindgut (incl. rectum) were sampled, while for *S. typhle*, fore-, mid- and hindgut (incl. rectum) were chosen (Fig. 1). Even though all samples GIT segments appeared to be empty during dissection, it cannot be fully excluded that some portions of the allochthonous microbiota have lingered across samples. Samples were stored at −80 °C until DNA extraction. For histology only (Fig. 1), two additional individuals per species were dissected and GIT segments of one individual were fixed with 4% PFA (in PBS), while the other individual’s GIT segments were moved to 1% glutaraldehyde in 200 mM HEPES, pH 7.4, overnight. PFA-fixed samples were gradually moved to 70% EtOH, embedded in paraffin, sectioned (10 µm), and stained with an H&E stain using standard protocols. The glutaraldehyde-fixed samples were washed and post-fixed with 1% osmium tetroxide in 1.5% potassium ferricyanide for 1 hour on ice, washed repeatedly, incubated with 2% aqueous uranyl acetate for 1.5 hour at room temperature in the dark, dehydrated gradually with ethanol and moved to acetone. Samples were then gradually moved to epon, polymerized at 65 °C for 48 h, after which ultra-thin sections of 1 µm were prepared and these were stained with Richardson’s solution (azur II and methylene blue).

### DNA extraction, 16S rRNA gene V3–V4 sequencing and analysis

DNA from samples stored at −80 °C was extracted using the Qiagen DNeasy Blood & Tissue kit according to manufacture recommendations including a pre-treatment for Gram-positive bacteria (as in [22]). Approximately the same amount of total DNA (host DNA plus bacterial DNA) per sample was then used for library preparation and sequencing (V3–V4 region; forward primer “341f“:5’ → 3’: CCTACGGGNGGCWGCAG; reverse primer “806 R“: 5’ → 3’: GGACTACHVGGGTWTCTAAT [29, 30]); on Illumina MiSeq sequencers. As the amount of input DNA was mostly comprised of host DNA and the ratio of microbial to host DNA in the input DNA likely varied among samples, microbial community results derived from these samples likely reflect the microbial community of a similarly sized piece of host tissue and not a similar number of bacteria from a tissue. During library preparation samples may have been subjected to different PCR cycle numbers and thus relative abundance values cannot necessarily be compared (but see below). Obtained read count files were demultiplexed and adapter sequences were trimmed in Qiime (v. 2020.8.0; Fig. S1A [31]). Data were denoised, merged, chimeric reads were removed and reads were trimmed using DADA2 in Qiime (similarly to [22]; Fig. S1B). Taxonomy was assigned using the silva database (v.138SSUnr99-304.806 [32]), and mitochondria and chloroplast reads were removed. The dataset consisting of 2005 ASVs and 66 individuals (some with very low read count, see below) was than imported into R using the qiime2R package, where subsequent analyses were conducted (v.0.99.6 [33, 34]).

### Quantification of bacterial DNA via qPCR

To independently approximate the relative bacteria abundance in GIT segment samples used for the microbiota analysis, qPCR was performed using the same total extracted DNA (containing microbial and host DNA) across all samples on an Analytic Jena qTower^3^G thermocycler, using the BioRad iQ SYBR Green Supermix in a 20 µl reaction volume. Primer pairs targeting the 16S rRNA gene’s V3–V4 region did not yield acceptable qPCR efficiencies and melt curves, rendering them unreliable. Instead, qPCR was performed with the primer pair “E1052“(TGCATGGYTGTCGTCAGCTCG) and “E1193“(CGTCRTCCCCRCCTTCC), targeting the gene’s V7 region. Using the same amount of input total DNA and a final concentration of 80 nM for forward and reverse primer, C_t_ values were determined (Supplementary Data 1 [35]). Melt curves were inspected to confirm amplification of a single target. Samples were run in triplicates and outlier samples were determined by being more than one CT off the median sample of the triplicate. Such outliers were excluded, as were samples without an obtained CT value (CT > 40). Samples for which more than one replicate had to be excluded this way were repeated and only considered for analyses when at least two replicates were then retained (*n* = 61). All data were obtained from four qPCR plate runs, and an additional six samples for standardizing across plates were also run in triplicates per plate (*E. coli* DNA with known concentrations from 25ng/µl to 0.25pg/µl). Differences in these standards’ average CT values per plate were used to correct sample CT values for plate effects. Additionally, NTC samples suggested that contamination/background amplification started at CTs > 27, which thus should be interpreted as no evidence of microbiota within the actual sample.

### Statistical analyses

Total read sums for each sample were calculated and they varied substantially, with one sample not obtaining any reads. Shannon alpha diversity was calculated, excluding the sample without reads (package “vegan” v.2.6 [36]). To gauge if samples with low read counts reliably reflected the alpha diversity correctly, for four samples with varying total read count sums an additional analysis was conducted: per sample, Shannon alpha diversity was calculated using all available reads, after which an increasing percentage of randomly chosen reads were excluded, each time followed by a calculation of the alpha diversity (Fig. S2). These analyses suggest that alpha diversity values are very robust against small numbers of reads per sample. Indeed, only samples with fewer than 100–200 reads are expected to show substantially underestimated alpha diversities. This data set contains eight such samples (one of which with zero reads), four of which belonging to *G. aculeatus* foregut samples, one to a hindgut sample, and the remaining two to *S. typhle* hindgut samples. A linear mixed model was used to examine the relationship between individuals’ C_t_ values and their read count sums, using the function lme(), with a random intercept for “fish id” and a variance structure with “varIdent(form = 1~|segment*species)” (*n* = 61; package “nlme” v.3.1 [37]). Three additional similar linear mixed models using either “read count sums” (*n* = 66), “ct values” (*n* = 61) or “alpha diversity” (*n* = 65) as independent variable and “species” and “GIT segment”, their interaction, and “sex” as dependent variables were fitted. The models also included a variance structure accounting for heteroscedasticity across GIT segment levels interacting with species, and random intercepts accounting for samples coming from the same individual. Based on these models the following nine pairwise comparisons were calculated per model and *p*-values were corrected via the “fdr” method (package: “multcomp” v.1.4 [38]): *G. aculeatus* stomach vs. foregut, *G. aculeatus* stomach vs. hindgut, *G. aculeatus* foregut vs. hindgut, *G. aculeatus* stomach vs. *S. typhle* foregut, *G. aculeatus* foregut vs. *S. typhle* foregut, *G. aculeatus* hindgut vs. *S. typhle* hindgut, *S. typhle* foregut vs. midgut, *S. typhle* foregut vs. hindgut, *S. typhle* midgut vs. hindgut (see Supplementary Data 2 for full model results). As “sex” was never having a significant effect, this factor was not considered for these comparisons (so, 0.5 in contrasts matrix). Another linear mixed model was used to assess the effect of read count sums on alpha diversity: alpha diversity was treated as dependent variable and read count sums, species, and their interaction as independent variables. Also, random intercepts were fitted for each fish individual. ANOVA type 3 was used to obtain an ANOVA table for the model, including *p*-values (package “car” v.3.1 [39]; see Supplementary Data 2 for full model results).

**Fig. 2:**
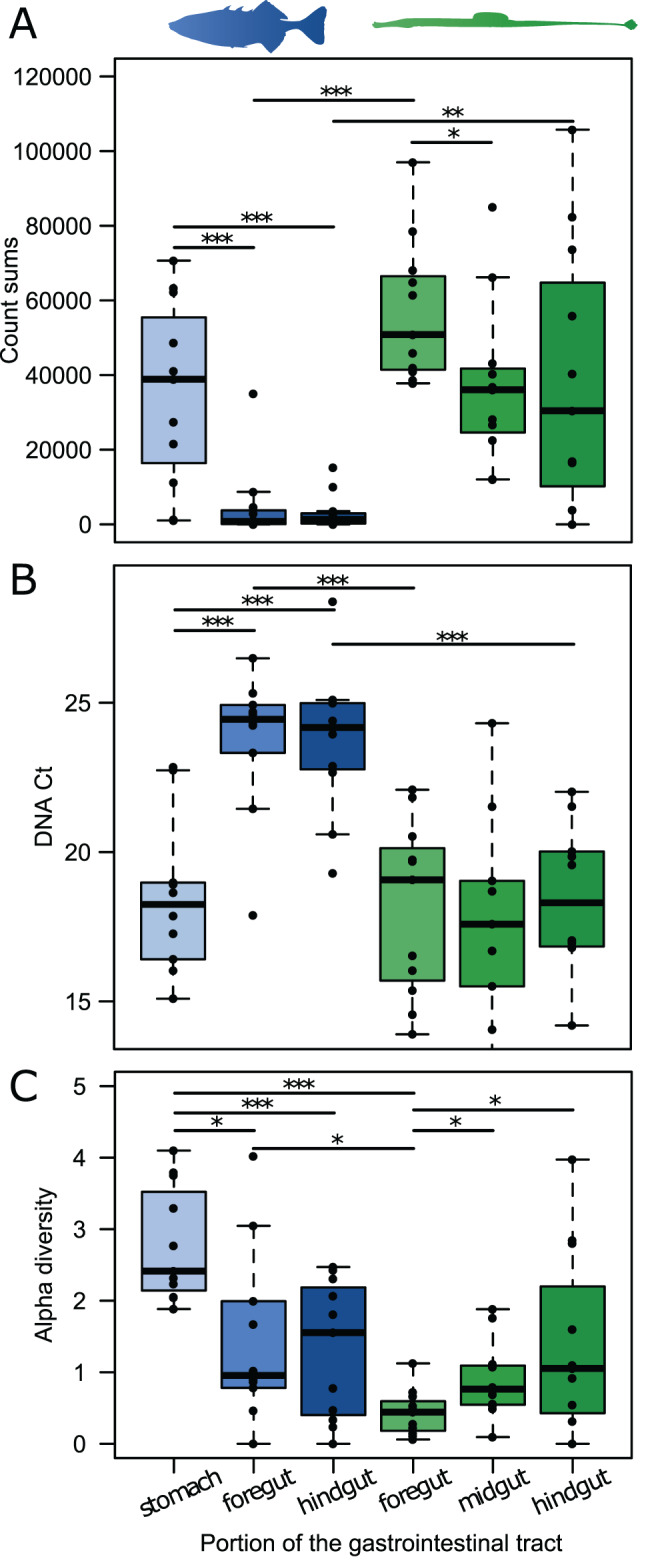
Microbial abundance & diversity in the gastrointestinal tracts of *S. typhle* & *G. aculeatus.* Microbial abundances in the stomach, foregut and hindgut of *G. aculeatus* (blue) and foregut, midgut and hindgut of *S. typhle* (green) deduced from microbiota filtered read counts (**A**) and qPCR analyses (**B**). Alpha diversity of both *G. aculeatus* and *S. typhle* across the same segment of the gastrointestinal tract (**C**). Horizontal bars indicate significantly different group means suggested by mixed linear models after fdr correction (*p* < 0.05)

For further analyses, read counts belonging to taxa of the same genus were pooled, and if only higher taxonomic level membership was available, this was used. Microbiota ASVs with taxa “unassigned” or “uncultured” were excluded as they could not be pooled into meaningful taxonomic groups subsequently. Additionally, samples with very few counts were removed from the analysis (<147 counts, see Fig. 2&S2) as their reads likely only insufficiently represent real microbiota composition. Finally, one *G. aculeatus* midgut sample was excluded for being a strong outlier, leaving a total of 59 samples (at least three samples per sex and subgroup) and 132 taxonomic groups. An abundance-aware jaccard dissimilarity matrix was calculated on read counts and variance across the six sample groups was found to not be different when “individual” was considered as strata (using the betadisper() function, package ‘vegan’, v.2.6–8; F = 0.8336, df = 5, *p* = 0.53). PerMANOVA using this dissimilarity matrix as dependent data, species, GIT segment and their interaction as independent variables, and the individual as strata was performed (using function adonis2(); package ‘vegan’). For 13 selected groups an indicator species analysis on the count matrix was performed (function multcomp(…, duleg = F, restcomb = selected_groups, func=”r.g”, control = how(nperm = 999)) from package ‘indicspecies’; v.1.8.0 [40]), which were: (1–6) for both species each GIT segment by itself, (7) both anterior segments, (8–9) both species’ fore- or hindgut segments together, (10) all *G. aculeatus* segments together, (11) all *S. typhle* segments together, (12) all mid- and hindgut segments of both species together, and (13) all segments of both species together except *G. aculeatus* stomach. Note that resulting *p*-values are not corrected for multiple testing. An nMDS-analysis on the same dissimilarity matrix as for the PerMANOVA was performed for two dimensions, as further dimensions only marginally reduced ordination stress. Contributions of all indicator species were projected onto the ordination (function metaMDS(); package ‘vegan’).

## Results

Gross morphological observation of the GIT in *G. aculeatus* and *S. typhle* confirmed the expected differences in the GIT: while *G. aculeatus* has a distinct stomach posterior to the esophagus, enveloped by a thick zone of muscles, no such structure could be found in *S. typhle* (Fig. 1). Instead, we assumed the stomach was lost in *S. typhle* and did not evolutionarily evolve into a foregut. Thus, its anterior segment of the gut (foregut) was treated as homolog to the most anterior gut segment posterior to the stomach in *G. aculeatus*. Finally, both species possess a somewhat visually distinct hindgut.

Bacterial 16S-rRNA gene sequencing revealed strongly varying total count sums across samples. As the same amount of total extracted DNA (fish + bacterial DNA) was used as an input for library preparation, this suggests that the abundance of bacteria cells relative to host cells varied across samples. However, varying PCR cycles during library preparation may have contributed to this pattern. QPCR using 16s-rRNA gene primers on extracted total DNA was performed and confirmed that read sums from amplicon sequencing are highly significantly linked with bacterial DNA quantities, i.e., the higher the read sums the lower the C_t_ values (χ = 102.98, df = 1, *p* < 0.001; Fig. 1). Both individuals’ read count sums and C_t_ values, i.e., the measures of bacterial DNA abundance, suggest that the stomach in *G. aculeatus* contains significantly higher relative amounts of bacterial DNA (compared to host DNA) than the front- and hindgut, which do not differ from each other (Supplementary Data 1, Supplementary Data 2; Fig. 2A, B). In contrast, in *S. typhle* only the read count sums suggest that the midgut segments had just significantly fewer reads than the foregut, while the hindgut did not differ significantly, possibly also due to its high variance, and C_t_ values did not show any difference among GIT segments. Additionally, pair-wise comparisons of read count sums and C_t_ values between species in front- and hindgut segments were significantly higher (i.e., indicating more bacteria) in *S. typhle* compared to their counterpart in *G. aculeatus* (Fig. 2A, B). Interestingly, the stomach of *G. aculeatus* and the foregut of *S. typhle* did not differ in read count sums or C_t_ values, suggesting similar bacteria abundance.

In *G. aculeatus*, alpha diversity was highest in the stomach, while fore- and hindgut did not differ from each other. However, as some gut-segments in *G. aculeatus* had very low sequencing depth (Fig. 2A), alpha diversity might be slightly underestimated (Fig. S2). In *S. typhle*, the opposite pattern was found, with the foregut showing the lowest alpha diversity and more posterior segments not differing. When comparing the two species, *G. aculeatus*’ stomach and foregut showed a higher alpha diversity than the foregut of *S. typhle*, although this was less pronounced for the foregut-to-foregut comparison (Fig. 2C). As this already suggests, alpha diversity was significantly affected by total read count sums (*p* = 0.020) and by its interaction with species (*p* = 0.004), with read numbers affecting diversity positively in *G. aculeatus* but negatively in *S. typhle* (Supplementary Data 2). Additionally, our subsampling analyses illustrates that low sequencing depth (i.e., low total read sums) affected alpha diversity estimates likely only in very few samples (Fig. S2).

**Fig. 3:**
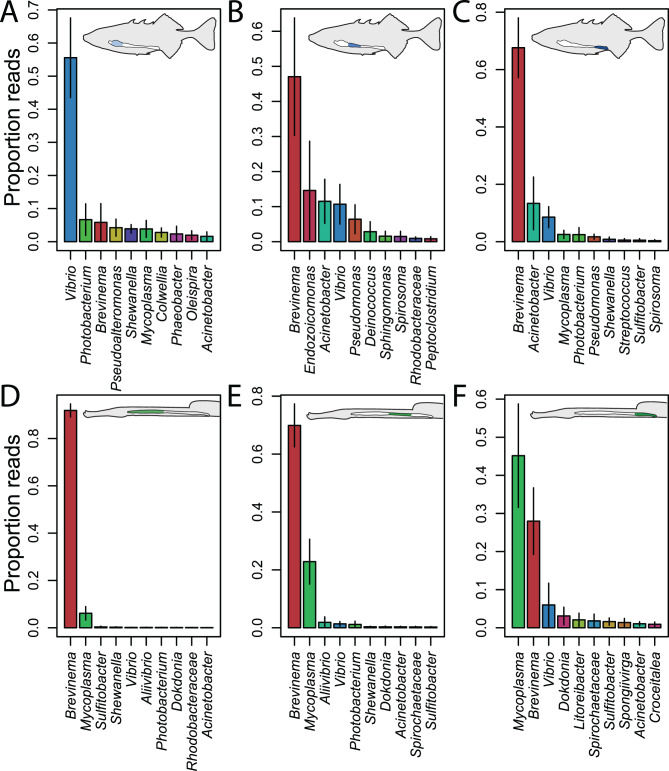
Microbial genera’s abundances across gastrointestinal tracts of *G. aculeatus* and *S. typhle.* Proportions of read counts across the ten most common bacterial genera in *G. aculeatus’* stomach (**A**), foregut (**B**) and hindgut (**C**), as well as in *S. typhle*’s foregut (**D**), midgut (**E**) and hindgut (**F**). Error bars represent standard errors

Bacteria genera’s contributions to the microbiota composition vary between species and across GIT segments (see also indicator species analysis below). In *G. aculeatus*, the stomach is clearly dominated by *Vibrio* (~56%), while no other genus contributes more than 7%. These others, however, contribute relatively evenly, as reflected in a relatively high alpha diversity for this GIT segment (see also Fig. 2C). *G. aculeatus*’ fore- and hindgut were dominated by *Brevinema*, *Acinetobacter* and also *Vibrio*, while the foregut also featured *Endozoicomonas* as a major contributor (Fig. 3B, C). In *S. typhle* fewer genera dominate the three considered gut segments: in the foregut *Brevinema* dominates with almost 92% of total reads and *Mycoplasma*, with ~6% of reads, being the only other genus being represented with more than 1%. The midgut shows a pattern intermediate to fore- and hindgut (Fig. 3E) and in the hindgut *Mycoplasma* (45%) overtook *Brevimena* (28%) as the most abundant genus, and also seven other of the ten most contributing genera contributed more than 1% of total reads, including *Vibrio* (~6%), *Dokdonia* (~3), *Litoreibacter* (~2%), *Spirochaetacea* (genus annotation not available; ~2%), *Sulfitobacter* (~2%), *Spongiivirga* (~1%) and *Acinetobacter* (~1%) (Fig. 3F; microbial taxa contributions on family, order, class and phylum level are available in Fig. S3-6). On a higher phylogenetic level this translates to members of the *Proteobacteria* dominating the stomach of *G. aculeatus*, while members of *Spirochaetota* increasingly dominating in more posterior gut segments. In *S. typhle*, members of *Spirochaetota* dominate the fore- and midgut, while in more posterior gut segments members of *Firmicutes* (which eventually are most abundant on average), *Proteobacteria* and *Becteroidota* all contribute substantially (Fig. S6).

**Fig. 4:**
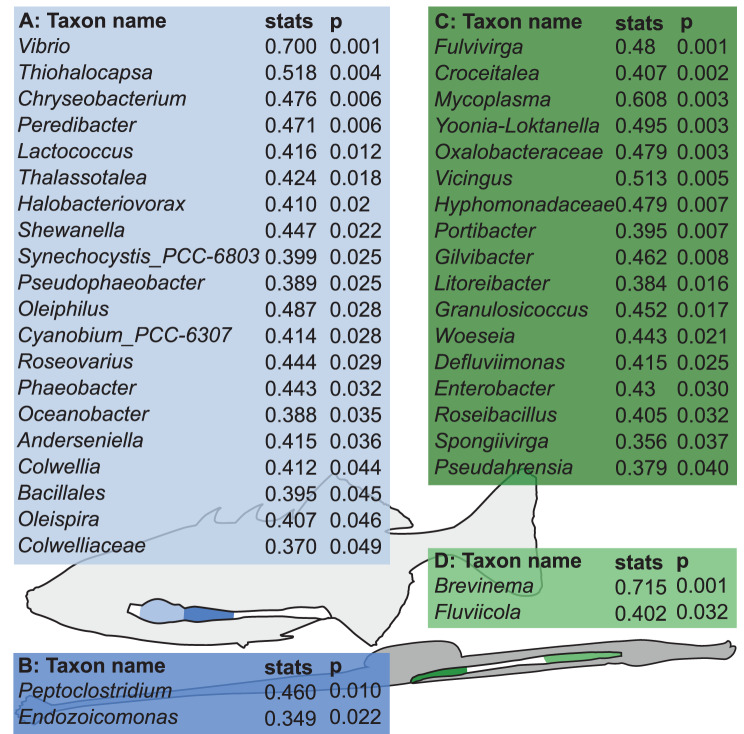
Microbial taxa indicative for segments of the gastrointestinal tract. Indicator analysis on a genus-level count data matrix. Most indicator taxa (20) were found for the stomach in *G. aculeatus* (**A**), while only two taxa being indicative for its hindgut (**B**). In *S. typhle*, 17 taxa were indicative for the hindgut (**C**) while 2 were indicative for its hindgut (i.e., *S. typhle* as a species; **D**)

**Fig. 5:**
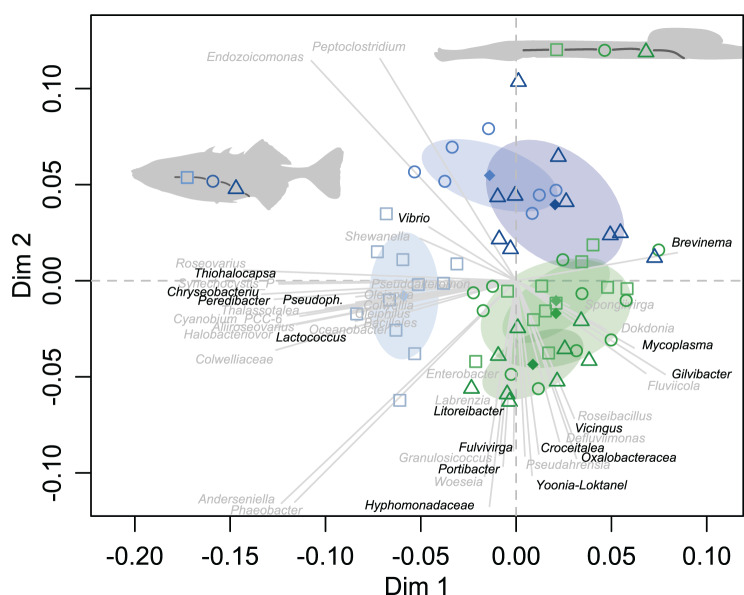
NMDS sample scores and microbial taxa indicating GIT segments. NMDS analysis suggests a pronounced differentiation between microbiota composition of *G. aculeatus*’ stomach and other samples (dim 1), and further distinction between *S. typhle*’s samples and those of *G. aculeatus* (dim 2). Shown microbial taxa are projections of significant (0.05 > *p* > 0.01; in grey) or highly significant (*p* < 0.01; in black) indicator taxa from corresponding analyses. Ellipses are 0.5 CI for GIT segments and species. Rectangles are anterior segments, circles midsegments and triangles posterior segments (=hindguts). *G. aculeatus* samples are shown in blue shades while *S. typhle* is shown in green shades

A PerMANOVA revealed that the microbiota compositions of the two species are indeed significantly different (F = 11.9, df = 1, *p* = 0.002), that they also differ among GIT segments (F = 3.5, df = 2, *p* = 0.002) and that these two variables interact significantly (F = 3.9, df = 2, *p* = 0.002; Supplementary Data 2). Furthermore, the microbiota of the two sexes differed in composition (F = 0.68, df = 1, *p* < 0.001) and the sex effect on the microbiota composition depended on the species (F = 1.26, df = 1, *p* < 0.001). Microbial taxa indicative of certain GIT segment groups (see Materials & Methods section) were identified using indicator species analysis, which revealed for four of the investigated groups significant indicator taxa. Most of these were identified for the stomach segment of *G. aculeatus* (20 taxa) and the hind gut of *S. typhle* (17 taxa), while the fore gut segment in *S. typhle* (2 taxa) and the mid gut in *G. aculeatus* (2 taxa) had only few taxa assigned to them as indicators (Fig. 4). Group differentiation was visualized using an nMDS ordination, with indicator taxa projected onto it (Fig. 5). Taken together the nMDS and indicator species analysis, *G. aculeatus*’ stomach and *S. typhle’s* hindgut are most distinct from other GIT segments.

## Discussion

In vertebrates, stomachs play crucial roles in digestion and as gatekeepers for microbes entering the gastrointestinal tract. Lineages with secondary loss of the stomach, such as several agastric actinopterygians (e.g., some Tetraodontiformes, Cypriniformes, Beloniformes and Syngnathiformes), compensate for these important functions leaving the immunological and physiological consequences for these lineages largely unknown to date [41].

In our study, we investigated the microbial abundance within the gastrointestinal tract in a pair of sympatric coastal fishes with and without a stomach. Our data suggests that the agastric *S. typhle* has a significantly higher microbiota abundance in the gut than the gastric *G. aculeatus*, while the stomach of the latter shows microbial abundance comparable to gut segments of *S. typhle*. We acknowledge that investigating only species with similar (but not identical) trophic niches cannot provide irrefutable evidence (see also [41]), but our analysis suggests that the stomach significantly decreased bacterial abundance in *G. aculeatus*, in line with its hypothesized function as an immunological gate-keeper [1, 2]. While this stands in contrast to several previous studies suggesting that gut-regions contain higher bacterial abundances compared to the stomach (e.g. [42, 43]), these studies did not indicate if only autochthonous or also the allochthonous (i.e., transient) microbiota was considered. Additionally, often only culturable bacteria were investigated, which constitute only a small and non-representative proportion of the actual bacterial diversity, especially considering the low pH requirements of the stomach autochthonous microbiota [44]. Evolutionarily losing the suggested gatekeeper function of the stomach thus likely affected the whole GIT microbiome and likely the host’s immune-homeostasis, as more transient microbial taxa are allowed to proceed into the gut. Interestingly, we found that the stomach in *G. aculeatus* harbored highly diverse microbiota – higher than the subsequent foregut segment, but also compared to the foregut segment in *S. typhle*, which held a similar bacterial abundance (Fig. 2). As expected, the stomach of *G. aculeatus* is the most distinct sample in our study. Using taxonomically related bacteria with known ecological niche to estimate ecological niches, this GIT segment harbored indicative taxa associated with the more acidic pH expected from vertebrate stomachs, such as *Lactococcus*, but also several motile predatory strains, such as *Peredibacter* and *Halobacteriovorax*, likely reflecting the high bacteria (and thus prey cell) density in this GIT segment. Furthermore, several indicator lineages with members known to decompose organic matter, such as *Colwelliaceae*, *Oleispira* or *Shewanella*, may have a mutualistic relationship with the host by assisting in chyme digestion [45–48]. *Vibrio* was identified as the most significant indicator lineage, reflecting its abundance in the stomach of *G. aculeatus*, and many of its lineages act as opportunistic pathogens [28, 49–51]. Stomachs have been reported to retain food chyme for some time after feeding and stomach-specific microbial composition may thus partially be shaped by food items (and their corresponding allochthonous microbiota) which could affect our results [52]. However, examined individuals were starved for 24 hours before sampling to reduce the contribution of allochthonous microbes and during dissection no food items have been found in the stomach of these fish, which makes us confident that this effect is unlikely to explain the observed patterns. Our data suggests the microbial diversity of *G. aculeatus*’ stomach was thus exceptionally high, and while subsequent fore- and hindgut sections showed lower alpha diversity, at least the foregut appeared to be still more diverse than its counterpart in *S. typhle*, although both feature two indicator taxa. Generally, this highlights how in *G. aculeatus* microbial abundance overall appeared to be lower but microbial diversity higher, while in *S. typhle* microbial abundance appeared to be much higher (except when compared to the stomach), but diversity not (and it could even be lower) compared to *G. aculeatus*. This may indicate that, in *G. aculeatus*, relatively low bacterial abundances coincided with a more diverse microbiota, whereas in *S. typhle* high bacterial abundance was not associated with increased diversity. One possible interpretation is that elevated bacterial abundance could place greater demands on the host immune system, potentially increasing energetic costs and affecting health. Alternatively, the modified immune system of *S. typhle* (and many other syngnathids) may allow them to tolerate higher bacterial abundances in their GIT without said costs [18–20]: the loss of MHCII as well as other immune genes, and the functional loss of the spleen (and potentially the gut-associated lymphoid tissue [53]); may affect the tolerance towards microbes in the GIT, contributing to increased or altered overall microbiome patterns.

One peculiarity of *S. typhles’* hindgut microbiota our data suggested was the genus *Mycoplasma* (they are not absent from *G. aculeatus*, but exceedingly rare). Its abundance increased from anterior (~6% of reads) to posterior (~45% of reads), which is noteworthy, as *Mycoplasma* lineages have been described as likely mutualistic partners in salmonids (which have a stomach [54]), although many lineages can also have a pathogenic lifestyle [55]. As such, their abundance is possibly affected by nutrition and may compensate partially for the likely reduced digestive capabilities of the agastric pipefish GIT (Jin et al., 2019). *Fulvivirga*, an indicator taxon for the hindgut, in which also *Mycoplasma* is most abundant, has also been described to comprise lineages able to enzymatically degrade a number of complex carbohydrates, such as alginate, chitin and starch [56], presenting them also as lineages with potentially mutualistic function. Other chemoheterotrophs, which may act as mutualistic partners during digestion, include *Portibacter*, *Gilvibacter*, *Granulosicoccus* and *Woesia*. This surprising diversity of putatively mutualistic bacteria suggests that stomach loss may facilitated the gut’s colonization/maintenance of bacteria populations assisting digestion, which could be an advantage counterbalancing the loss of the stomach’s gatekeeper function and increased immune vulnerability. More specifically, a study in the herbivore fish *Kyphosus sydneyanus* showed highest alpha diversity in the hindgut compared to more anterior gut segments and the hindgut was dominated by the phylum Bacteroidota, some Firmicutes and few Proteobacteria – a pattern more similar to the hindgut of *S. typhle* than any other GIT segment studied here (Fig. S3 [12]). In *K. sydneyanus*, further evidence was collected to link this microbiota to fermentation, and given the similarity to the hindgut microbiota of *S. typhle* and the surprisingly high amount of vegetation in the food of *S. typhle* (at least in some populations [57–59]), it is plausible that *S. typhle*’s hindgut may support microbes fermenting gut content (e.g., such as the indicator lineage *Enterobacter*), which might facilitate an at least partial digestion of plant matter. Note, however, that our functional/ecological interpretations of microbes remain speculations as they are based on higher taxonomic levels (genus/family) and may therefore not reflect the actual bacterial strain represented in the samples.

Overall bacteria abundances in this study suggest that only very few lineages dominated in most GIT segments, which stands in some contrast to previous studies from other locations, where lineage contributions were more even (e.g. [60]). As habitats and the food items therein have a dominant influence on microbiota compositions of fish [60–62], we did not expect strong overlap with such previous studies, but rather conclude that these divergences are likely the result of the unique microbiome of the Baltic Sea. Furthermore, it remains to be assessed whether this difference was due to us feeding our fish with commercially bought mysids, which likely brought their own microbiota, affecting the autochthonous microbiota, or whether this decrease in diversity was due to the Baltic Sea’s unique ecology.

Finally, the derived and unusual immune system of *S. typhle* may have contributed to its unique microbiota assemblage: these fish lack MHCII and several other immune genes, which may affect its tolerance towards microbes in its GIT when compared to taxa with more conventional immune gene repertoires [9, 19, 20]. Intestine microbiota have been linked to affect especially the innate immune system in fish [63], but the adaptive immune system may act as an (at least weak) ecological filter for the GIT [64]. Whether or not these immunological quirks do actually change immune cell activity and thus potentially the immunological tolerance towards (certain) microbes is unknown to date, as major immune cell types appear to be present in *S. typhle* and more in-depth investigations are still ongoing ([65] *and personal communication with the author J. Parker*). Ultimately, we cannot assess in our system if immunological alterations may have contributed to observed patterns. In light of the immunological oddity of *S. typhle*, the fact that only two species were compared, and considering aforementioned limitations of our microbiota analyses, we encourage further studies with additional species pairs of sympatric gastric and agastric fish to examine if patterns found in this study indeed reflect a wider evolutionary pattern. Additionally, examinations of individuals taken directly taken from their natural habitat would bridge this study’s gap to the actual ecologically-relevant natural microbiome of these fish.

## Conclusions

In vertebrates, stomachs improve digestion rates by chemically breaking down food items and prevent most ingested microbes to enter the intestines by partially sterilizing food particles. The paradox of repeated stomach loss across major teleost lineages remains unresolved to date and studying only one species pair naturally cannot fully uncover the immunological and physiological consequences distinguishing both phenotypes, necessitating further research on additional species pairs and/or artificially created agastric individuals, e.g., via gene knockouts [41]. Nonetheless, studying the gastrointestinal microbiota of two similarly-sized, sympatric fish species allowed us to shed light on the possible drivers of underlying evolutionary process and their trade-offs: gastric fish may have increased chemical digestion capability and effective pathogen exclusion from the more vulnerable intestines. In contrast, stomach loss may have facilitated increased microbial diversity with the potential for more effective mutualistic relationships between host and microbes (e.g., via fermentation) at the cost of reduced chemical digestion and increased vulnerability to pathogens [66].

## Electronic supplementary material

Below is the link to the electronic supplementary material.

Supplementary material 1

Supplementary material 2

Supplementary material 3

## Data Availability

The dataset supporting the conclusions of this article is available in the NCBI repository, bioproject PRJNA1303079, https://www.ncbi.nlm.nih.gov/bioproject/PRJNA1303079/. Additional datasets supporting the conclusions of this article are included within the article Supplementary Data 1, Supplementary Data 2 and Supplementary Figures.

## Abbreviations

Fig.: Figure
GIT: Gastrointestinal tract
G.: *Gasterosteus*
S.: *Syngnathus*
qPCR: Quantitative Polymerase Chain Reaction
nMDS: Non-metric MultiDimensional Scaling
PerMANOVA: Permutational Multivariate ANalysis Of VAriance
