# Supplementary material for: Gastrointestinal microbiota of sympatric pipefish (Syngnathus typhle) and stickleback (Gasterosteus aculeatus) indicate trade-off associated with evolutionary stomach loss
Source: BMC Ecol Evol. 2026 Jun 30;26:56. doi: 10.1186/s12862-026-02546-4 (PMC13344009; doi:10.1186/s12862-026-02546-4)
Supplement: Supplementary file 2 — Supplementary material 2 [file 12862_2026_2546_MOESM2_ESM.rtf]

Abbreviations:“spec” = species factor with levels “Gac” for G.aculeatus and “Sty” for S.typhle“tis” = tissue (= GIT segment) factor with levels “a” (most anterior) to “c” (most posterior). Note that levels a, b & c in Gas are stomach, foregut and hindgut, respectively, but in Sty these are foregut, midgut & hindgut.“id” = individual identifiers factor“sex” = individuals’ sexes (Female or Male)“reads" = vector with read sums per individual and GIT segment“ct” = vector with Ct values per individual and GIT segment“test.frame” = data frame in which used data vectors were combined for analyses via lme()“con1” = contrasts matrix with specified pairwise comparisonsProvided is the R output # CT values are linked with individuals’ read count sums> mod_dt_rc<-lme(reads~ct ,random=~1|id, method="REML", data=test.frame,weights=varIdent(form=~1|tis*spec),na.action="na.omit")> Anova(mod_dt_rc)Analysis of Deviance Table (Type II tests)Response: reads    Chisq Df Pr(>Chisq)    ct 102.98  1  < 2.2e-16 ***# Analysis of read count sums as a function of GIT segment and species> mod_rc<-lme(reads~spec*tis+sex ,random=~1|id, method="REML", data=test.frame,weights=varIdent(form=~1|tis*spec),na.action="na.omit")> mod_rcLinear mixed-effects model fit by REML  Data: test.frame   Log-restricted-likelihood: -665.2161  Fixed: reads ~ spec * tis + sex  (Intercept)      specSty         tisb         tisc      sexMale specSty:tisb specSty:tisc    33861.193    21689.636   -30310.364   -31922.000     2482.313    10593.182    13714.909 Random effects: Formula: ~1 | id        (Intercept) ResidualStdDev:    1.307154 24932.42Variance function: Structure: Different standard deviations per stratum Formula: ~1 | tis * spec  Parameter estimates:    a*Gac     b*Gac     c*Gac     a*Sty     b*Sty     c*Sty 1.0000000 0.4002290 0.2026128 0.7797203 0.8641450 1.4598690 Number of Observations: 66Number of Groups: 22 > Anova(mod_rc, type="II")Analysis of Deviance Table (Type II tests)Response: reads           Chisq Df Pr(>Chisq)    spec     34.6232  1  4.001e-09 ***tis      21.9391  2  1.722e-05 ***sex       0.9477  1     0.3303    spec:tis  1.0994  2     0.5771    ---Signif. codes:  0 ‘***’ 0.001 ‘**’ 0.01 ‘*’ 0.05 ‘.’ 0.1 ‘ ’ 1> summary(glht(mod_rc, linfct=con1),test=adjusted("fdr"))	 Simultaneous Tests for General Linear HypothesesFit: lme.formula(fixed = reads ~ spec * tis + sex, data = test.frame,     random = ~1 | id, weights = varIdent(form = ~1 | tis * spec),     method = "REML", na.action = "na.omit")Linear Hypotheses:                               Estimate Std. Error z value Pr(>|z|)    tisb == 0                        -29069       8197  -3.546 0.001172 ** tisc == 0                        -30681       7775  -3.946 0.000358 ***tisb_tisc == 0                    -1612       3372  -0.478 0.711803    specSty == 0                      22931       9617   2.384 0.030800 *  tisb_specSty == 0                 52000       6589   7.892  2.6e-14 ***tisc_specSty,tisc == 0            35404      11080   3.195 0.003141 ** specSty_specSty,tisb == 0        -19717       8750  -2.253 0.036343 *  specSty_specSty,tisc == 0        -18207      12442  -1.463 0.184319    specSty,tisb_specSty,tisc == 0     1510      12753   0.118 0.905742    ---Signif. codes:  0 ‘***’ 0.001 ‘**’ 0.01 ‘*’ 0.05 ‘.’ 0.1 ‘ ’ 1(Adjusted p values reported -- fdr method)# Analysis of Ct values as a function of GIT segment and species> mod_ct<-lme(ct~spec*tis+sex ,random=~1|id, method="REML", data=test.frame,weights=varIdent(form=~1|tis*spec),na.action="na.omit")> mod_ctLinear mixed-effects model fit by REML  Data: test.frame   Log-restricted-likelihood: -147.9063  Fixed: ct ~ spec * tis + sex  (Intercept)      specSty         tisb         tisc      sexMale specSty:tisb specSty:tisc   19.3591645    0.2172727    5.2345455    4.5338101   -0.8484683   -4.7918182   -3.8418101 Random effects: Formula: ~1 | id        (Intercept) ResidualStdDev:    2.388686 1.559587Variance function: Structure: Different standard deviations per stratum Formula: ~1 | tis * spec  Parameter estimates:       a*Gac        b*Gac        c*Gac        a*Sty        b*Sty        c*Sty 1.0000000000 1.4119073656 2.2010319722 0.0001584889 1.3021098534 2.6502363630 Number of Observations: 64Number of Groups: 22 > Anova(mod_ct, type="II",na.action="na.omit")Analysis of Deviance Table (Type II tests)Response: ct           Chisq Df Pr(>Chisq)    spec     21.9703  1  2.769e-06 ***tis      18.0148  2  0.0001225 ***sex       0.0474  1  0.8275627    spec:tis 13.7206  2  0.0010486 ** ---Signif. codes:  0 ‘***’ 0.001 ‘**’ 0.01 ‘*’ 0.05 ‘.’ 0.1 ‘ ’ 1> summary(glht(mod_ct, linfct=con1),test=adjusted("fdr"))	 Simultaneous Tests for General Linear HypothesesFit: lme.formula(fixed = ct ~ spec * tis + sex, data = test.frame,     random = ~1 | id, weights = varIdent(form = ~1 | tis * spec),     method = "REML", na.action = "na.omit")Linear Hypotheses:                               Estimate Std. Error z value Pr(>|z|)    tisb == 0                       5.24010    1.13637   4.611 1.17e-05 ***tisc == 0                       5.26012    1.03835   5.066 3.66e-06 ***tisb_tisc == 0                  0.02002    1.09842   0.018    0.985    specSty == 0                   -0.66138    1.39936  -0.473    0.946    tisb_specSty == 0              -5.62450    1.23448  -4.556 1.17e-05 ***tisc_specSty,tisc == 0         -5.35939    1.14459  -4.682 1.17e-05 ***specSty_specSty,tisb == 0      -0.27698    1.37587  -0.201    0.946    specSty_specSty,tisc == 0       0.28513    1.14987   0.248    0.946    specSty,tisb_specSty,tisc == 0  0.56211    1.37431   0.409    0.946   ---Signif. codes:  0 ‘***’ 0.001 ‘**’ 0.01 ‘*’ 0.05 ‘.’ 0.1 ‘ ’ 1(Adjusted p values reported -- fdr method)# Analysis of alpha diversity as a function of GIT segment and species> mod_di<-lme(div~spec*tis+sex ,random=~1|id, method="REML", data=test.frame,weights=varIdent(form=~1|tis*spec),na.action="na.omit")> mod_diLinear mixed-effects model fit by REML  Data: test.frame   Log-restricted-likelihood: -75.7486  Fixed: div ~ spec * tis + sex  (Intercept)      specSty         tisb         tisc      sexMale specSty:tisb specSty:tisc    2.6886378   -2.3522811   -1.3209775   -1.4729429    0.1782661    1.7725462    2.4152633 Random effects: Formula: ~1 | id         (Intercept)  ResidualStdDev: 0.0001012596 0.7393238Variance function: Structure: Different standard deviations per stratum Formula: ~1 | tis * spec  Parameter estimates:    a*Gac     b*Gac     c*Gac     a*Sty     b*Sty     c*Sty 1.0000000 1.7014045 1.3189997 0.4550639 0.7392891 1.7455199 Number of Observations: 65Number of Groups: 22 > Anova(mod_di, type="II")Analysis of Deviance Table (Type II tests)Response: div           Chisq Df Pr(>Chisq)    spec     67.4035  1  < 2.2e-16 ***tis       2.1530  2     0.3408    sex       1.4057  1     0.2358    spec:tis 26.6750  2  1.613e-06 ***---Signif. codes:  0 ‘***’ 0.001 ‘**’ 0.01 ‘*’ 0.05 ‘.’ 0.1 ‘ ’ 1> summary(glht(mod_di, linfct=con1),test=adjusted("fdr"))	 Simultaneous Tests for General Linear HypothesesFit: lme.formula(fixed = div ~ spec * tis + sex, data = test.frame,     random = ~1 | id, weights = varIdent(form = ~1 | tis * spec),     method = "REML", na.action = "na.omit")Linear Hypotheses:                               Estimate Std. Error z value Pr(>|z|)    tisb == 0                      -1.23184    0.46087  -2.673  0.02256 *  tisc == 0                      -1.38381    0.37655  -3.675  0.00107 ** tisb_tisc == 0                 -0.15197    0.49472  -0.307  0.85355    specSty == 0                   -2.26315    0.25619  -8.834  < 2e-16 ***tisb_specSty == 0              -1.03130    0.41059  -2.512  0.02703 *  tisc_specSty,tisc == 0          0.06298    0.48770   0.129  0.89725    specSty_specSty,tisb == 0       0.45157    0.19352   2.333  0.02943 *  specSty_specSty,tisc == 0       0.94232    0.40211   2.343  0.02943 *  specSty,tisb_specSty,tisc == 0  0.49075    0.42256   1.161  0.31563    ---Signif. codes:  0 ‘***’ 0.001 ‘**’ 0.01 ‘*’ 0.05 ‘.’ 0.1 ‘ ’ 1(Adjusted p values reported -- fdr method)# Analysis of diversity as a function of read count and species> mod_div<-lme(div~spec*reads ,random=~1|id, method="REML", data=test.frame[which(!is.na(test.frame[,5])),])> mod_divLinear mixed-effects model fit by REML  Data: test.frame[which(!is.na(test.frame[, 5])), ]   Log-restricted-likelihood: -113.9268  Fixed: div ~ spec * reads   (Intercept)       specSty         reads specSty:reads  1.579329e+00 -2.123873e-01  1.946311e-05 -3.005244e-05 Random effects: Formula: ~1 | id         (Intercept) ResidualStdDev: 5.354178e-05  1.00395Number of Observations: 65Number of Groups: 22 > Anova(mod_div, type="III")Analysis of Deviance Table (Type III tests)Response: div              Chisq Df Pr(>Chisq)    (Intercept) 52.9151  1  3.483e-13 ***spec         0.2837  1    0.59431    reads        5.3941  1    0.02021 *  spec:reads   8.0822  1    0.00447 ** ---Signif. codes:  0 ‘***’ 0.001 ‘**’ 0.01 ‘*’ 0.05 ‘.’ 0.1 ‘ ’ 1# PerMANOVA on log-transformed reads# Sample sizes> table(species,tissue,sex_nmds), , sex_nmds = Female       tissuespecies a b c    Gac 5 3 5    Sty 5 5 4, , sex_nmds = Male       tissuespecies a b c    Gac 6 4 5    Sty 6 6 5# Distance matrix> dist<-vegdist(t(log(counts1+1)), distance="jaccard")# Variance across groups not different> anova(betadisper(dist,group=as.factor(paste(species,tissue,sex_nmds))))Analysis of Variance TableResponse: Distances          Df  Sum Sq  Mean Sq F value Pr(>F)Groups    11 0.13102 0.011910  1.0911 0.3888Residuals 47 0.51305 0.010916# PerMANOVA> adonis2(t(counts1)~species*tissue*as.factor(sex_nmds), distance="jaccard", strata=fish.id_nmds, by="terms")Permutation test for adonis under reduced modelTerms added sequentially (first to last)Blocks:  strata Permutation: freeNumber of permutations: 999adonis2(formula = t(counts1) ~ species * tissue * as.factor(sex_nmds), by = "terms", strata = fish.id_nmds, distance = "jaccard")                                   Df SumOfSqs      R2       F Pr(>F)    species                             1   4.0898 0.19367 17.1701  0.001 ***tissue                              2   2.1059 0.09972  4.4205  0.001 ***as.factor(sex_nmds)                 1   0.1614 0.00765  0.6778  0.001 ***species:tissue                      2   2.4324 0.11519  5.1060  0.001 ***species:as.factor(sex_nmds)         1   0.2982 0.01412  1.2519  0.001 ***tissue:as.factor(sex_nmds)          2   0.3360 0.01591  0.7053  0.851    species:tissue:as.factor(sex_nmds)  2   0.4981 0.02359  1.0456  0.492    Residual                           47  11.1951 0.53015                   Total                              58  21.1170 1.00000                ---Signif. codes:  0 ‘***’ 0.001 ‘**’ 0.01 ‘*’ 0.05 ‘.’ 0.1 ‘ ’ 1
