# Supplementary material for: Gastrointestinal microbiota of sympatric pipefish (Syngnathus typhle) and stickleback (Gasterosteus aculeatus) indicate trade-off associated with evolutionary stomach loss
Source: BMC Ecol Evol. 2026 Jun 30;26:56. doi: 10.1186/s12862-026-02546-4 (PMC13344009; doi:10.1186/s12862-026-02546-4)
Supplement: Supplementary file 3 — Supplementary material 3 [file 12862_2026_2546_MOESM3_ESM.pdf]

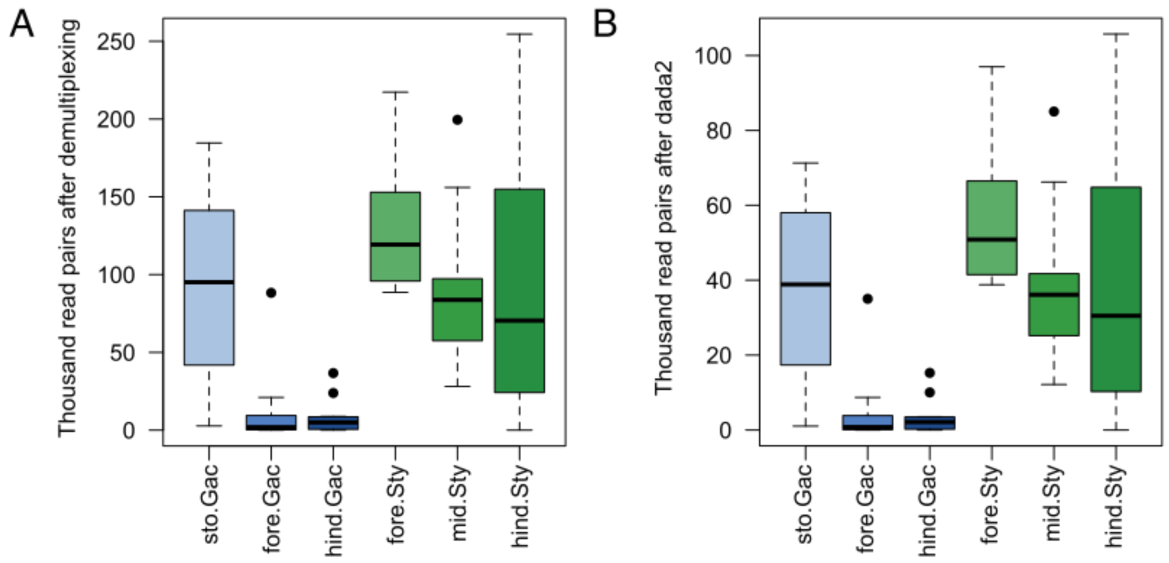

Fig. S1: **Read count pairs after demultiplexing and after DADA2 pipeline for *G. aculeatus* and *S. typhle***
Read pairs per sample, treatment and species after demultiplexing (A) and after the DADA2 pipeline concluded (B). Gac = G. aculeatus, Sty = S. typhle, sto=stomach, fore=foregut, mid=midgut, hind=hindgut.

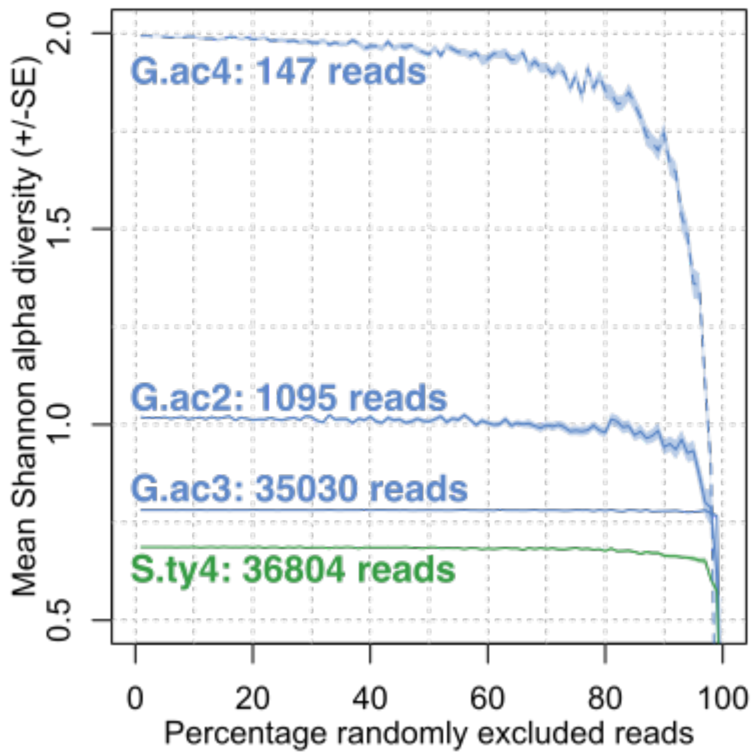

Fig. S2: **Mean Shannon alpha diversity in four samples when read numbers are randomly decreased**
Mean Shannon alpha diversity values calculated for four selected samples when a portion of reads was randomly excluded from the respective sample (mean of 50 iterations, +/- SE). Samples comprised three *G. aculeatus* (G.ac) foregut segments with very low (G.ac4), low (G.ac2) and relatively high (Gac3) read counts and a sample with the average read count in *S. typhle* midgut (S.ty4), which is comparable to the highest read count among G. aculeatus foregut samples (G.ac3=35030, S.ty4: 36804). Alpha diversity measures appear stable even when large portions of their actual reads are excluded randomly. Only samples with very low read counts (e.g. G.ac4) may likely not accurately reflect the samples actual Shannon alpha diversity, although deviations are likely minor for samples retaining at least 100-200 reads.

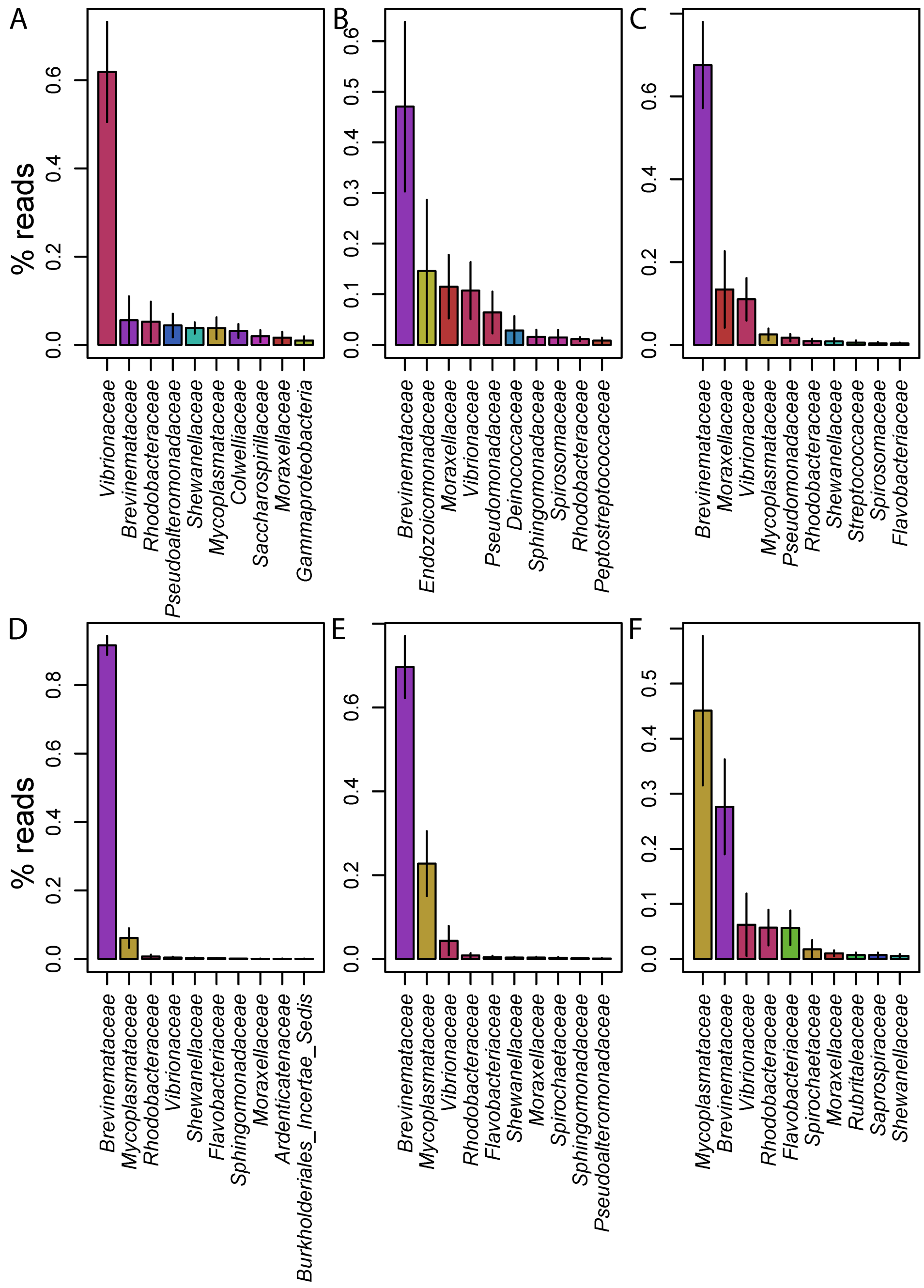

Fig. S3: **Microbial families' abundances across gastrointestinal tracts of *G. aculeatus* and *S. typhle***

Proportion of read counts across the ten most common microbiome families in *G. aculeatus'* stomach (A), foregut (B) and hindgut (C), as well as in *S. typhle*'s foregut (D), midgut (E) and hindgut (F). Error bars represent standard errors.

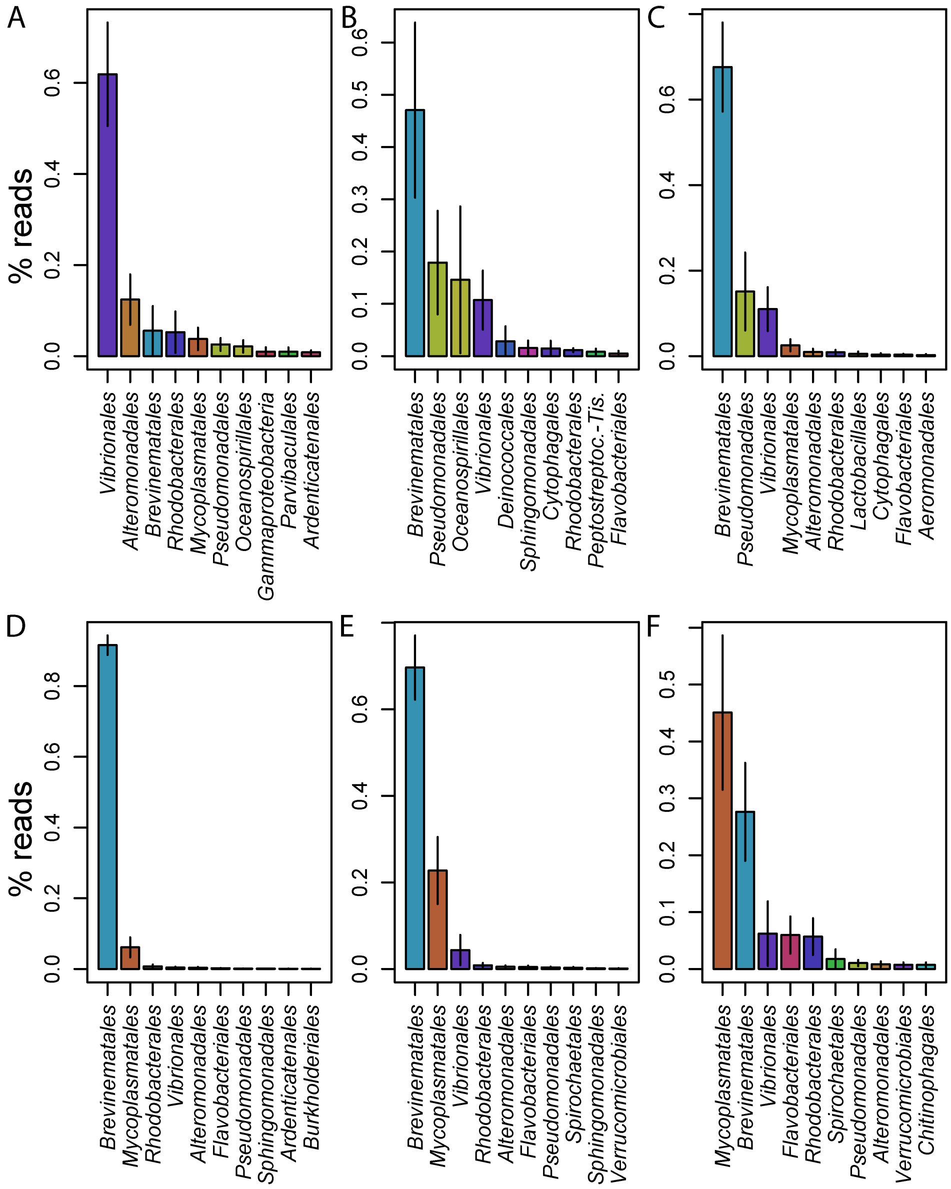

Fig. S4: **Microbial orders' abundances across gastrointestinal tracts of *G. aculeatus* and *S. typhle***

Proportion of read counts across the ten most common microbiome orders in *G. aculeatus'* stomach (A), foregut (B) and hindgut (C), as well as in *S. typhle*'s foregut (D), midgut (E) and hindgut (F). Error bars represent standard errors.

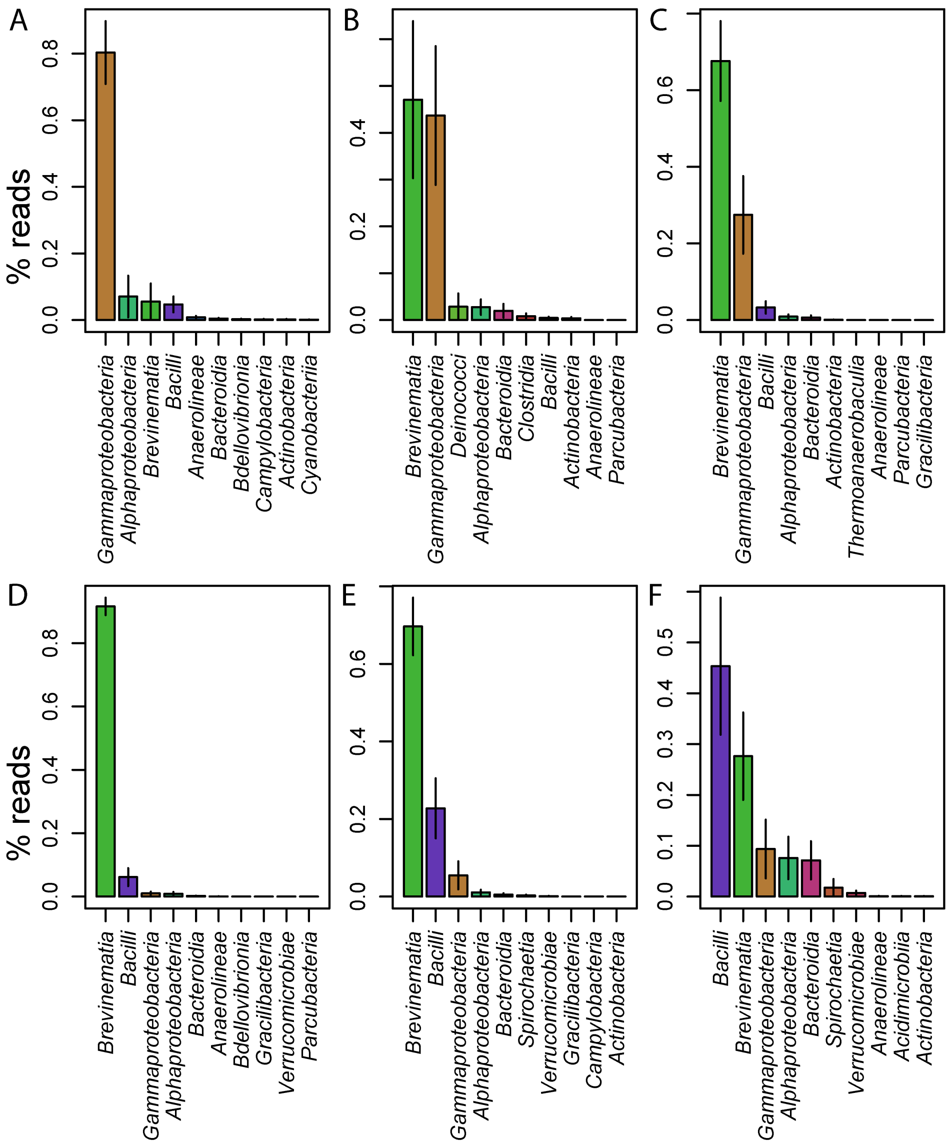

Fig. S5: **Microbial classes' abundances across gastrointestinal tracts of *G. aculeatus* and *S. typhle***

Proportion of read counts across the ten most common microbiome classes in *G. aculeatus'* stomach (A), foregut (B) and hindgut (C), as well as in *S. typhle*'s foregut (D), midgut (E) and hindgut (F). Error bars represent standard errors.

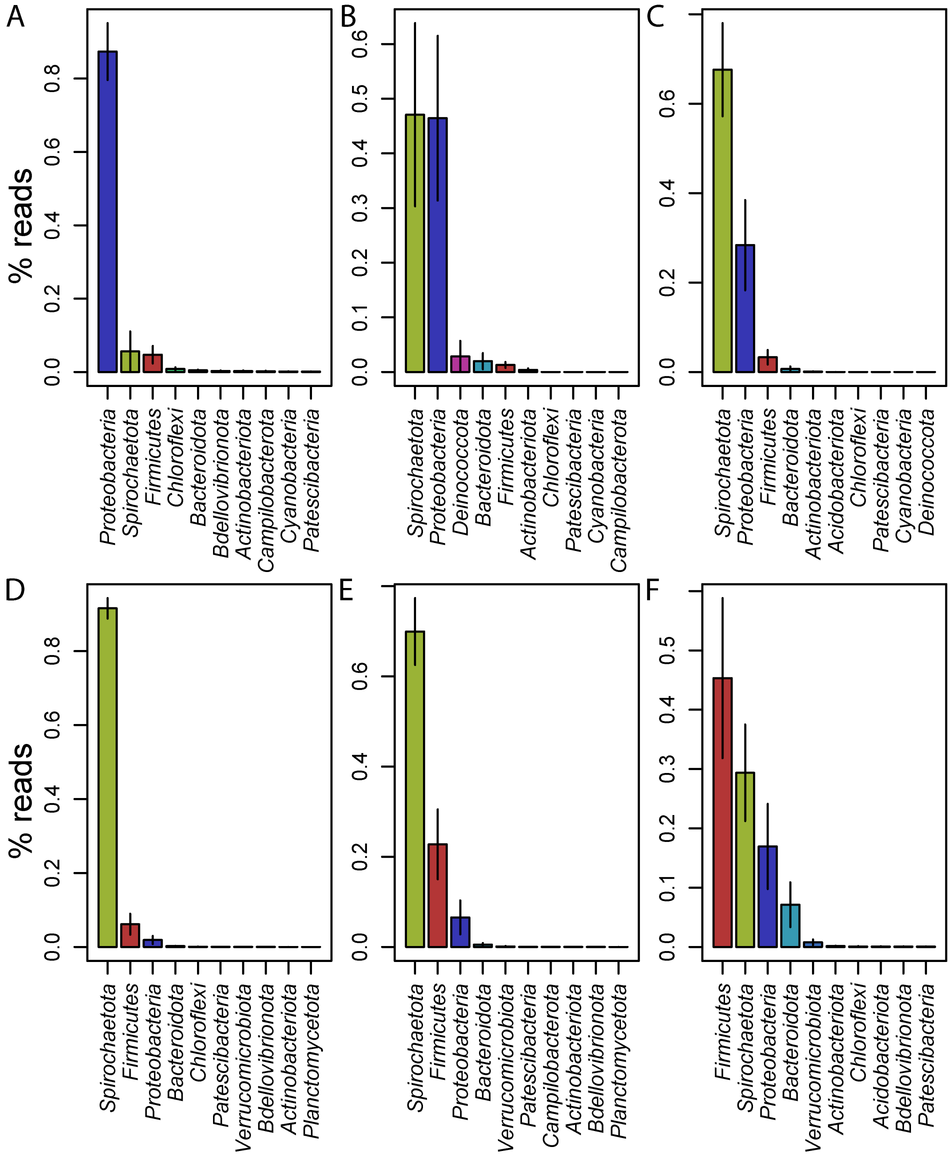

Fig. S6: **Microbial phyla's abundances across gastrointestinal tracts of *G. aculeatus* and *S. typhle***

Proportion of read counts across the ten most common microbiome phyla in *G. aculeatus'* stomach (A), foregut (B) and hindgut (C), as well as in *S. typhle*'s foregut (D), midgut (E) and hindgut (F). Error bars represent standard errors.
